# On the Pro-Metastatic Stress Response to Cancer Therapies: Evidence for a Positive Co-Operation between TIMP-1, HIF-1α, and miR-210

**DOI:** 10.3389/fphar.2012.00134

**Published:** 2012-07-12

**Authors:** Haissi Cui, Sebastien Grosso, Florian Schelter, Bernard Mari, Achim Krüger

**Affiliations:** ^1^Klinikum Rechts der Isar der Technischen Universität München, Institut für Experimentelle Onkologie und TherapieforschungMünchen, Germany; ^2^Institut de Pharmacologie Moléculaire et Cellulaire, UMR 6097 CNRS/UNSASophia Antipolis, France

**Keywords:** HIF-1a, microenvironment, microRNA, miR-210, MMP, pro-metastatic, TIMP-1, Met

## Abstract

In contrast to expectations in the past that tumor starvation or unselective inhibition of proteolytic activity would cure cancer, there is accumulating evidence that microenvironmental stress, such as hypoxia or broad-spectrum inhibition of metalloproteinases can promote metastasis. In fact, malignant tumor cells, due to their genetic and epigenetic instability, are predisposed to react to stress by adaptation and, if the stress persists, by escape and formation of metastasis. Recent recognition of the concepts of dynamic evolution as well as population and systems biology is extremely helpful to understand the disappointments of clinical trials with new drugs and may lead to paradigm-shifts in therapy strategies. This must be complemented by an increased understanding of molecular mechanism involved in stress response. Here, we review new roles of Hypoxia-inducible factor-1 (HIF-1), one transcription factor regulating stress response-related gene expression: HIF-1 is crucial for invasion and metastasis, independent from its pro-survival function. In addition, HIF-1 mediates pro-metastatic microenvironmental changes of the proteolytic balance as triggered by high systemic levels of tissue inhibitor of metalloproteinases-1 (TIMP-1), typical for many aggressive cancers, and regulates the metabolic switch to glycolysis, notably *via* activation of the microRNA miR-210. There is preliminary evidence that TIMP-1 also induces miR-210. Such positive-regulatory co-operation of HIF-1α, miR-210, and TIMP-1, all described to correlate with bad prognosis of cancer patients, opens new perspectives of gaining insight into molecular mechanisms of metastasis-inducing evasion of tumor cells from stress.

## Introduction

Late stage cancer with occurrence of metastasis is the greatest and most challenging problem for research aimed at the development of new therapeutic concepts and agents against malignant tumors. In fact, most cancer patients die of metastasis and not of the primary tumor (Sporn, 1996). Current therapies are genotoxic-based DNA-damaging agents and cell cycle inhibitors and show some efficacy in early stages of cancer only (Yamaguchi and Perkins, 2012). However, in the cell population of a progressing tumor, these therapies often select for clones which, due to their genetic and epigenetic instability, accumulate mutations which abrogate checkpoint functions and apoptosis-inducing programs (Yamaguchi and Perkins, 2012). This is the major obstacle for complete eradication of most disseminated cancers (Gatenby, 2009).

As an alternative, more recent therapy strategies were based on the increased knowledge of the specific molecular biology of tumor cells.

Two prominent examples are the anti-angiogenic and anti-proteolytic therapy approaches which aimed at depriving the tumor cells from the line of supply of oxygen and nutrients or tools needed to invade the surrounding tissue, respectively. Promising target proteins were the vascular endothelial growth factor (VEGF) as a crucial promoter of new vessel formation during angiogenesis (Ferrara, 2002) as well as matrix metalloproteinases (MMPs) as important enzymes with the ability to degrade virtually all components of the extracellular matrix (Egeblad and Werb, 2002; Overall and López-Otin, 2002). These approaches initially were found to be very effective in pre-clinical settings as they led to marked inhibition or even shrinkage of tumors (Zucker et al., 2000; Ferrara et al., 2004). However, application of the respective inhibitors in clinical settings provided either disappointing results in the case of anti-angiogenic therapies (Cai et al., 2011) or even failure of clinical trials in the case of MMP inhibition (Zucker et al., 2000). Indeed, it already became evident in pre-clinical experiments that inefficacy or even detrimental effects of these therapy approaches can arise, especially in the context of late stage cancer with metastasis. While primary tumors may be inhibited by anti-angiogenic (Casanovas et al., 2005; Ebos et al., 2009; Pàez-Ribes et al., 2009) or anti-proteolytic (Della Porta et al., 1999) therapies, both drastically promoted tumor cell invasion and metastasis (Della Porta et al., 1999; Krüger et al., 2001; Casanovas et al., 2005; Ebos et al., 2009). Notable examples are the induction of liver metastasis by the hydroxamate-like synthetic inhibitor of matrix metalloproteases batimastat (Krüger et al., 2001) as well as findings that VEGFR/PDGFR kinase inhibitors or VEGFR-specific antibodies increase multi-organ metastasis (Ebos et al., 2009) or metastasis to the liver and lymph nodes, respectively (Pàez-Ribes et al., 2009).

The main explanation of these “surprises” relies on the too long ignored appreciation that tumors represent an eco-system of heterogeneous tumor and host cell populations, where mechanisms of evolutionary dynamics and population biology apply, leading to the conclusion that over-simplistic interference will yield unforeseen and even detrimental effects (Gatenby, 2009). In addition, it is necessary to more carefully analyze the complex interdependencies of targeted gene products including the activity and expression of connected proteins and its effect on signal transduction and cell behavior, in other words the systems biology of specific therapeutic interference (Krüger, 2009; Krüger et al., 2010; Sela-Passwell et al., 2011). With such mind-set it was possible to solve the paradox why elevated systemic levels of tissue inhibitor of metalloproteinases-1 (TIMP-1), i.e., correlate with bad prognosis of many cancers (Zucker et al., 2000). There is data suggesting that broad-spectrum inhibition of MMPs by TIMP-1 interferes with the negative regulation of the MET tyrosine kinase by blocking the MET-shedding activity of ADAM-10 (Kopitz et al., 2007). MET activity is a key promoter of the invasive growth program in tumor cells (Boccaccio and Comoglio, 2006). In addition, broad-spectrum inhibition of MMPs leads to an evasive shift of proteolytic activity from the inhibited MMPs toward serine proteases such as the urokinase-type plasminogen activator (uPA) in the microenvironment, which was associated with a drastic promotion of tumor cells to metastasize (Kopitz et al., 2007). Such complexities and evolutionary adaptations of tumor cells or, as in the above case of the tumor microenvironment, are extremely relevant not only for therapeutic inhibition of MMPs but for all attempts to inhibit proteases as “obvious” tools for tumor cell invasion and metastasis (Krüger et al., 2010).

Such Darwinian selection of tumor cells with a more aggressive phenotype can also be the cause of problems with anti-angiogenic therapy (Gatenby and Gillies, 2004; Michieli, 2009). Inhibition of angiogenesis leads to hypoxia which was initially thought to lead to suffocation of tumor cells and subsequent total elimination of the tumor (Hanahan and Folkman, 1996; Dang and Semenza, 1999). However, malignant tumors meet the hypoxic challenge not only by induction of angiogenesis. Instead, in fast-growing neoplasias hypoxia selects for malignant tumor cells which are adapted to hypoxia by using a gene expression program allowing a metabolic switch from dependence on oxygen for energy generation to energy production under anaerobic conditions by glycolysis only (Gatenby and Gillies, 2004). In fact, malignant tumor cells are even able to leave the site if unfavorable conditions persist by initiating an invasive growth program (Pennacchietti et al., 2003). This program is highly dependent on signaling of the MET tyrosine kinase receptor (Pennacchietti et al., 2003) and ultimately leads to metastasis, the main cause of death for cancer patients.

The ability of tumor cells to respond to stress by inducing angiogenesis, performing glycolysis, and initiating the invasive growth program has to be maintained throughout the steps of the metastatic cascade (Gatenby and Gillies, 2004). Hypoxia-inducible factor-1 (HIF-1) is a transcription factor which seems to be crucial for all the above-mentioned stress responses of malignant tumor cells.

In this review we propose a molecular mechanism from available data which explains how metastasis formation can be driven by an increase in stress on tumor cells by anti-angiogenic and anti-proteolytic agents. We summarize evidence for and propose a new context between one of the most central regulators of the proteolytic network, TIMP-1, the major regulator of tumor cell stress response, HIF-1, and its main downstream microRNA (miRNA) effector, miR-210.

## Hif-1α Regulates Response of Tumor Cells to Stress

**Figure 1:**
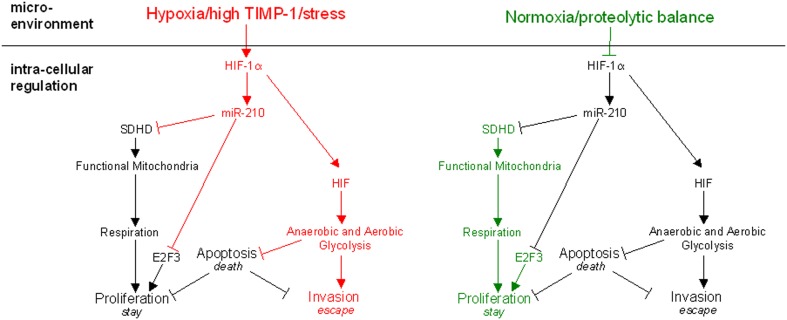
**The proposed model of molecular mechanisms regulating the three choices of tumor cells under stress**. Microenvironmental factors regulate the activity of HIF-1α. In addition to normoxia or hypoxia, changes of the proteolytic network can also determine the response of the tumor cell. While normoxia and proteolytic balance favor proliferation (right panel), which reflects the adaption of the tumor cell *in situ*, hypoxia or alterations in the proteolytic network promote the induction of pro-evasive mechanisms (left panel). Respiration is impaired upon HIF-1α stabilization not only by hypoxia but also in the case of proteolytic imbalance imposed by high TIMP-1 levels or similar environmental stress. It leads to upregulation of miR-210 which directly inhibits expression of the subunit D of the succinate dehydrogenase complex (SDHD) and simultaneously induces cell cycle arrest by inhibition of E2F3. Thus, the tumor cell switches to the evasive metastatic phenotype for which adaptation to hypoxia by their potential of executing anaerobic glycolysis is a pre-requisite.

Competition with the surrounding tissue for nutrients, oxygen, and space disturbs the proteolytic balance and forces tumor cells to adapt, escape, or undergo apoptosis (Figure 1). Cell fate, i.e., whether a tumor cell succumbs to the stress by apoptosis (*death*), adapts by ensuring survival at the site (*stay*) or leaves in the search of better conditions (*escape*) is determined by HIF-1 dependent intracellular pathways (Figure 1). Under normoxic conditions HIF-1α, one of two subunits of HIF-1, is hydroxylated by iron- and oxygen-dependent prolyl-hydroxylases (Lando et al., 2002; Kaelin and Ratcliffe, 2008), modified by the E3 ubiquitin ligase von Hippel Lindau (Jaakkola et al., 2001), and subsequently degraded in the proteasome. A lack of oxygen renders the prolyl-hydoxylases inactive so that HIF-1α can associate with constitutively expressed HIF-1β and form the heterodimer HIF-1 (Majmundar et al., 2010). HIF-1 translocates to the nucleus, binds to hypoxia-response elements (HREs), and initiates transcription of target genes which allow adaptation to stress (Wenger et al., 2005).

HIF-1 can also be induced by hypoxia-independent factors (Wenger et al., 2005), such as inflammatory cytokines like IFN-γ and IL-4 (Majmundar et al., 2010) as well as NF-κB (Wilczynski et al., 2011). HIF-1α mRNA expression and protein stability are further upregulated by mTORC1 (Bernardi et al., 2006) and the PI3K pathway (Mottet et al., 2003), which can initiate a HIF-1-mediated response independent of hypoxic stress.

Recently, high level of metalloproteinase inhibitor TIMP-1 in the tumor microenvironment was discovered as a new inducer of HIF-1 in a liver metastasis model (Schelter et al., 2011b). Based on this observation, the induction of HIF-1α gene expression by TIMP-1 was further confirmed *in vitro*: Exposure of a lymphoma tumor cell line to recombinant TIMP-1 led to an increase in HIF-1α mRNA level, protein and the HIF-1α downstream target CA-9 (Schelter et al., 2011b). This observation represents a new connection between the major regulator of stress response in tumor cells, HIF-1, and TIMP-1, a central player of the proteolytic network. TIMP-1 determines tissue homeostasis (Krüger et al., 2010) and can, if systemically elevated, promote metastasis (Kopitz et al., 2007). As metastasis accounts for 90% of solid tumor dependent deaths (Gupta and Massagué, 2006), the induction of the metastatic potential of tumor cells by TIMP-1 and HIF-1 must be considered crucial for successful treatment of malignant tumors.

## Regulation of Tumor Cell Invasion by Hif-1

In addition to its critical role in tumor cell survival during hypoxic conditions in metastasis (Gatenby and Gillies, 2004), HIF-1 also directly promotes tumor cell malignancy by induction of an aggressive phenotype (Lu and Kang, 2010). HIF-1 leads to an upregulation of glycolysis while at the same time suppressing oxidative phosphorylation. Two consequences result from this metabolic adaptation: firstly, oxygen is no longer a limiting co-factor necessary for cellular energy supply. Secondly, lactate generated at the same time in large amounts can serve as a precursor for many metabolic intermediates needed for cell growth (Gatenby and Gillies, 2004). Secreted lactate can also be used by surrounding oxygenated tumor cells imported by the transporter MCT1, which in this context may be a possible therapeutic target (Sonveaux et al., 2008; Le Floch et al., 2011). Independence from oxygen and efficient lactate transport resourcefully exploit the available energy sources in the tumor (Sonveaux et al., 2008).

In order to balance the much less effective aerobic glycolysis, glucose uptake is increased by upregulation of glucose transporters like GLUT-1 by HIF-1 (Chen et al., 2001). In addition, a variety of pro-angiogenic factors including the VEGF (Forsythe et al., 1996) and PDGF (Bos et al., 2005) are upregulated by HIF-1 to ensure a sufficient supply of glucose and to escape the lack of oxygen resulting from the diffusion limit in solid fast-growing tumors.

Persistent lack of oxygen can ultimately lead to the selection of tumor cells which exhibit a more aggressive and invasive phenotype and allows escape from unfavorable conditions (Gatenby and Gillies, 2004). HIF-1 promotes metastasis by initiating epithelial-to-mesenchymal transition by the induction of the transcription factors Snail (Mak et al., 2010) and Twist (Yang et al., 2008), which in turn lead to a repression of E-Cadherin. Additionally, HIF-1 induces the expression of MET tyrosine kinase receptor (Pennacchietti et al., 2003) *via* HRE in the MET promoter region (Pennacchietti et al., 2003). MET-signaling in turn leads to an increase in cell motility and invasiveness which furnishes metastasis (Pennacchietti et al., 2003). Furthermore, HIF-1 promotes the expression of proteases enabling tumor cells to break through physical borders (Krishnamachary et al., 2003; Schelter et al., 2010).

The above-mentioned *in vitro* observations left it open, whether HIF-1α regulates tumor cell invasiveness directly *in vivo* and what molecular mechanisms determine direct pro-invasive functions of HIF-1 signaling (Bertout et al., 2008; Ruan et al., 2009). Until recently, it was hard to clarify this topic as the survival of tumor cells under microenvironmental stress usually depends on HIF-1 signaling (Semenza, 2003). Indeed, it could not be excluded that reduced metastasis upon HIF-1α depletion was simply a reflection of decreased survival (Schelter et al., 2010). Recently, this distinction was achieved using a hypoxia-tolerant tumor cell line, which does not rely on HIF-1 signaling for survival. It was shown that HIF-1 directly controls pro-invasive and pro-metastatic features of tumor cells independent of its promotion of cell viability (Schelter et al., 2010). Further, it was shown that HIF-1 plays a central role in the efficacy of metastasis formation and organ colonization by induction of MMP-9, one of the major gelatinases expressed by invasive tumor cells (Schelter et al., 2010). This separation of survival-dependent and independent HIF-1 effects allows differentiation between tumorigenic and metastasis-promoting effects of HIF-1, leading to a deeper insight into metastatic processes and stress response.

## HIF-Induced miR-210 and the Switch to Escape Mode

Recently, an additional level of HIF-dependent transcriptional regulation was elucidated, which involved HIF-induced up-regulation of miRNA (Kulshreshtha et al., 2007). MiRNAs function as components of ribonucleoprotein complexes referred as miRNA-induced silencing complexes (miRISCs). They primarily regulate gene expression through inhibition of RNA translation by binding to their target gene’s 3′ UTR. They also induce mRNA decay, resulting in the down-regulation of target mRNAs (Esquela-Kerscher and Slack, 2006; Ortholan et al., 2009; Fabian et al., 2010).

Upon hypoxia, microRNA miR-210 is induced by HIF-1 (Kulshreshtha et al., 2007). While a number of miRNAs are associated with stress response in tumor cells, miR-210 is the only “hypoxamir” which is most significantly upregulated in a number of cancers (Chan and Loscalzo, 2010). MiR-210 mediates a number of known HIF-1 downstream effects, most importantly the HIF-1 dependent suppression of oxidative phosphorylation (Chan et al., 2011).

Mechanistical insight into how HIF-1 inhibits mitochondrial respiration could be provided by studying the role of miR-210 in HIF-1 signal transduction: miR-210 regulates ISCU1/2 expression, a protein associated with iron metabolism and leads to its repression upon stress (Chan et al., 2009). In addition, miR-210 was shown to target Succinate Dehydrogenase Subunit D (Puisségur et al., 2011), a component of the mitochondrial complex II. This provides evidence that miR-210 mediates the functional loss of the respiratory chain, the metabolic switch induced in tumor cells upon hypoxic stress (Gatenby and Gillies, 2004).

Moreover, miR-210 is also involved in cell cycle by regulation of the E2F3 transcription factor: miR-210 mediates the suppression of E2F3 protein expression and might attenuate cell proliferation (Giannakakis et al., 2008; Puisségur et al., 2011).

In addition, a recent study showed that not only HIF-1 induces miR-210 transcription but that *vice versa* miR-210 stabilizes HIF-1α (Puisségur et al., 2011). The inhibition of miR-210 shortens the HIF-1 activity which indicates a positive auto-regulatory loop based on the suppression of the afore-mentioned Succinate Dehydrogenase Subunit D (SDHD) by miR-210 (Puisségur et al., 2011). Low level of Succinate Dehydrogenase subsequently leads to an accumulation of succinate which in turn inhibits HIF-1α prolyl-hydroxylases (Selak et al., 2005). An inhibition of HIF-1α regulating enzymes delays the degradation of HIF-1α and stabilizes HIF-1 (Puisségur et al., 2011). Thus, HIF-1, miR-210, and SDHD are not only connected by linear transcriptional regulation but are indeed involved in an auto-regulatory loop on protein level which prolongs HIF-1 activity and signaling.

In conclusion, the effects of miR-210 on proliferation and metabolism together with the stabilization of HIF-1 lead to an accentuation of tumor cell stress response (Figure 1). Malignant cells are selected for their ability to evade stress present in their microenvironment. They are able to do so as they adopt a more aggressive phenotype and increase their metastatic potential as suggested by a very recent study (Ying et al., 2011).

In addition to the effect of stress-induced expression of miR-210 on tumor cells, one could speculate that miR-210 is involved in the manipulation of pre-malignant tissue and modulation of the microenvironment at metastatic sites. To successfully colonize potential target tissue, tumor cells depend upon stromal and vascular cells (Gupta and Massagué, 2006). Hypoxic regions within metastases and high acidity in the tumor cell microenvironment stimulate HIF-1 dependent stress response not only in tumor cells but also in their direct microenvironment (Dayan et al., 2008). Induction of HIF-1 signaling and the effects of miR-210 might therefore not only promote an aggressive phenotype in tumor cells but also indirectly support metastasis by modulation of neighboring cells. This speculation is supported by the observation that forced expression of miR-210 in endothelial cells stimulated the formation of capillary-like structures and VEGF response and thus might promote angiogenesis upon hypoxia (Fasanaro et al., 2008).

It is also possible that not only hypoxia but also hypoxia-independent induction of HIF-1, e.g., by TIMP-1 (Schelter et al., 2011b), could stimulate miR-210 transcription, as we have preliminary evidence that exogenous TIMP-1 can lead to miR-210 upregulation in mouse livers.

## TIMP-1, A Possible Regulator of Pro-Evasive Stress Response?

TIMP-1 stands out as a soluble factor which does not only modulate the extracellular matrix by regulation of MMP-activity but as an inducer of HIF-1α, it also decides over cell fate by guiding tumor cells toward an invasive phenotype. The two mechanisms by which TIMP-1 interferes with the metastatic potential of a tumor cell can be demonstrated by the example of the HGF receptor MET. First, MET is stabilized on protein level by high TIMP-1 levels *via* the inhibitory effects of TIMP-1: as an inhibitor of ADAM-10, it prevents shedding of MET, leading to higher cell surface levels. This enables both the more effective signaling of HGF as well as HGF independent dimerization of MET which also results in increased signal transduction (Kopitz et al., 2007). Second, on a transcriptional level, TIMP-1 might activate HIF-1 which in turn upregulates the gene expression of MET by binding to the MET promoter (Pennacchietti et al., 2003).

A clue of the role of TIMP-1 in metastatic progression was recently shown utilizing a TIMP-1 knock down in tumor cells which significantly decreased tumor cell invasiveness and reduced the number of metastasis (Schelter et al., 2011a). Taken together with the newly discovered connection between TIMP-1 and HIF-1α expression, TIMP-1 evolves as an outstanding cutting point between stress response and the proteolytic network.

## Conclusion

Until recently TIMP-1 and HIF-1α (and in consequence miR-210) were seemingly totally un-related factors whose elevation is associated with bad prognosis for cancer patients (Zucker et al., 2000; Chan and Loscalzo, 2010; Wilczynski et al., 2011). In this review, we propose a functional pro-metastatic co-operation of these factors that link microenvironmental conditions to stress response involving the emerging field of gene regulation by miRNAs. Recent attempts to understand the complexity of system-wide cell signaling networks led to a deeper understanding of how self-contained networks can engage in crosstalk and contribute to cancer progression. The link between the proteolytic network and stress-related signaling by the natural broad-spectrum inhibitor TIMP-1 is one example of how interference with one network impacts on cell fate-decisions mediated by another pathway.

There is now increasing evidence that current experimental therapy approaches to eradicate the primary tumor may indeed lead to increased tumor cell aggressiveness. This aggressiveness, in turn, seems to be a natural adaptation strategy to environmental pressure known from all aspects of evolutionary selection. Future therapeutic targets should consider the context of both the flexibility and interplay of different balanced networks and the ability of tumor cells to adapt to environmental stress. Here, we evaluated recent evidence, of how changes in tissue homeostasis, e.g., by alteration in the proteolytic network can impact on metastasis by HIF-1 dependent increase in tumor cell invasiveness and aggressiveness. This link is provided by TIMP-1, which can elicit HIF-1-induced stress response (Schelter et al., 2010, 2011b) and lead to increased metastasis in addition to the pro-metastatic effects of TIMP-1 related to its inhibitory properties (Kopitz et al., 2007; Schelter et al., 2011a). Indeed, paradigm-shifts in the treatment of cancer (Gatenby, 2009), which include a more holistic appreciation of the underlying mechanisms seem to be necessary to overcome current problems in the field. Combinational therapy (Pennacchietti et al., 2003; Cunningham et al., 2011) or moderate treatments, aiming on containing the tumor instead of curing it (Gatenby, 2009), might overcome adaptation strategies of tumor cells. Anti-angiogenic reagents paired with inhibitors of MET-signaling are currently under test after the suggestion in 2003 (Pennacchietti et al., 2003; Michieli, 2009). Further, more knowledge on HIF-1 pathway inhibitors, including strategies targeting miRNAs (Medina and Slack, 2009) such as miR-210, might help to outwit tumor cells by removing the driving force of evolutionary pressure (Semenza, 2003; Lu and Kang, 2010; Wilczynski et al., 2011).

## Conflict of Interest Statement

The authors declare that the research was conducted in the absence of any commercial or financial relationships that could be construed as a potential conflict of interest.
